# Revealing the complexity of the epicardial secretome

**DOI:** 10.1038/s41598-025-24980-y

**Published:** 2025-11-21

**Authors:** Cláudia C. Oliveira, José Córdoba, John R. Pearson, Elizabeth Guruceaga, Ernesto Marín-Sedeño, María López-Moreno, Juan Antonio Guadix, Melissa García-Caballero, José M. Pérez-Pomares, Adrián Ruiz-Villalba

**Affiliations:** 1 Department of Animal Biology, Faculty of Sciences, University of Málaga, Campus de Teatinos s/n, 29080 Málaga, Spain; 2 Instituto de Investigación Biomédica de Málaga y Plataforma en Nanomedicina (IBIMA Plataforma BIONAND), Málaga, Spain; 3 Department of Molecular Biology and Biochemistry, Faculty of Sciences, University of Málaga, Campus de Teatinos s/n, 29080 Málaga, Spain; 4 Leica Microsystems, Barcelona, Spain; 5 Bioinformatics Platform, Center for Applied Medical Research (CIMA), University of Navarra, Pamplona, Spain; 6 Navarra Institute for Health Research (IdiSNA), Pamplona, Spain

**Keywords:** Developmental biology, Heart development

## Abstract

The epicardium, an epithelial layer covering the heart, plays pivotal roles in embryonic heart development and responses to adult cardiac damage. Epicardial-secreted molecular agents are known to be involved in the regulation of these phenomena, but how this regulation occurs is poorly understood. In this study, we have investigated extracellular vesicle (EV) and extracellular matrix (ECM) components of the epicardial secretome using a continuous mouse embryonic epicardial-derived cell (EPIC) line. Epicardial-derived EVs were isolated using differential ultracentrifugation from EPIC cultured at 1% (EVs-H1%), 5% (EVs-H5%), and 21% oxygen (EVs-N). EVs protein content was determined by tandem mass tag (TMT) proteomic analysis. The results showed that epicardial-derived EVs cargo is sensitive to the oxygen level of parenteral cells, increasing their content on glycolytic proteins as oxygen level decreases. Moreover, hypoxic-derived EVs were found to both increase EPIC proliferation and affect the metabolism of Human Umbilical Vein Endothelial Cells (HUVECs). On the other hand, epicardial-derived extracellular matrix (EPIC-ECM) was characterized by submitting decellularized EPIC to shotgun proteomics and comparing it to decellularized perinatal hearts and Matrigel®. We found that EPIC-ECM composition closely resembles that of embryonic cardiac tissue. Although the structural and basement membrane-associated proteins of EPIC-ECM were similar to those found in Matrigel®, EPIC-ECM exhibited higher protein diversity and was a more potent inducer of HUVEC proliferation. This work represents the first comprehensive and systematic proteomic analysis of two important components of the epicardial-derived secretome. Our experiments reveal that the epicardium responds to hypoxia by secreting EVs capable of modifying the metabolic responses of surrounding cells. Furthermore, EPIC-ECM promotes endothelial cell proliferation. These findings demonstrate the significant signaling abilities of the epicardial secretome and its potential contribution to cardiac development, both consistent with reports of endothelial responses following cardiac ischemic damage.

**Supplementary Information:**

The online version contains supplementary material available at 10.1038/s41598-025-24980-y.

## Introduction

The epicardium is the epithelium that covers the surface of the heart. Both cellular and molecular contributions from the epicardium are crucial for proper heart morphogenesis^1–4^. During embryonic development, some primitive embryonic epicardial cells activate an Epithelial-to-Mesenchymal Transition (EMT), giving rise to a population of highly migratory mesenchymal cells, named Epicardial-Derived Cells (EPDCs)^5–8^, which can differentiate into a myriad of cardiac cell types. In addition, epicardial epithelial cells and EPDCs secrete multiple instructive molecular signals including retinoic acid (RA), hepatocyte growth factor (HGF), vascular endothelial growth factor (VEGF), bone morphogenic proteins (BMPs), transforming growth factor-β (TGFβs), fibroblast growth factors (FGFs) and Insulin-like growth factor-1 (IGF1) among many other (see^9–18^). These molecules, which have mostly been regarded as paracrine signaling agents, are known to be indispensable for proper coronary vascularization^18–22^ and embryonic ventricular compact myocardium growth^18,23–26^. Accordingly, loss-of-function of specific epicardial genes such as Wilms’ tumor gene (*Wt1*), T-box transcription factor 18 (*Tbx18*)*,* or transcription factor 21 (*Tcf21*), results in severe defects in both coronary and chamber myocardial wall formation, leading to midgestational death^3,27,28^.

The role of the epicardium and EPDCs as sources of cells and paracrine signals in the developing heart has been extensively studied, overshadowing the critical contribution of the epicardium to the cardiac extracellular environment. First, the nascent extracellular matrix (ECM) deposited between the myocardium and the epicardium (known as subepicardium) is a low oxygen tension milieu that modulates EPDC differentiation, including coronary cell progenitors^27,29–32^. Furthermore, EPDCs are involved in interstitial ECM synthesis of the cardiac chambers, i.e. the ECM that accumulates between cardiomyocytes, a microenvironment critical for cardiac homeostasis^2^. Although the epicardium plays important roles in embryonic heart development and the onset and progression of adult heart disease, its contribution to cardiac ECM remains poorly characterized. Boezio and colleagues have demonstrated that the deletion of classical epicardial gene markers affects myocardial growth through the downregulation of ECM genes in zebrafish^33^ Accordingly, Sun and colleagues described the expression of dystroglican, a glycoprotein connecting the ECM with intracellular actin, in embryonic epicardial cells, and pointed out its relevance in epicardial EMT^34^.However, more research is required to clarify the specific contribution of the epicardial-derived ECM to heart development.

Another neglected element of the epicardial secretome is that of extracellular vesicles (EVs). EVs refers to non-replicative submicron lipid bilayer-enclosed particles that are released by virtually all cells^35^ and are heterogeneous in size and composition^36,37^. Due to the difficulty in defining EVs diversity, the current nomenclature for EVs is mainly based on their size (small or medium/large EVs) and on markers such as tetraspanins and other proteins related to the endosomal sorting machinery, which are helpful to identify specific EVs subtypes^37^. EVs are known to carry cellular-derived components such as nucleic acids, proteins, and lipids that are important players in cell signaling, both in health and disease^38^. Interestingly, it has been shown that EVs cargo composition correlates with the stimulus or biological condition triggering their formation and release^39,40^. These findings suggest the existence of intracellular selective cargo-sorting mechanisms^35,41^. Furthermore, it has been reported that EVs cargo can modulate the behavior of the recipient cell. For instance, cell preconditioning to hypoxia is known to result in the release of EVs that significantly improve cardiac performance under adverse conditions^42,43^. For this reason, EVs have gained much attention in the last decade^44^, having been proposed as a tool to diagnose and treat different diseases, including cardiovascular ones^45,46^. For instance, several studies have described the potential role of epicardial adipose tissue-derived EVs in the context of arrhythmias in adulthood^47–49^. However, no studies have been published regarding either the composition or the potential role of epicardial-derived EVs during the development of the heart.

In the present work, we have jointly characterized epicardial-secreted EVs and ECM proteins. To date, analysis of the epicardium-specific contribution to the cardiac extracellular environment during the development has remained elusive, mostly due to technical limitations in tracing the cell-specific origin of the secretome and the inherent challenges of isolating sufficient epicardial and EPDCs from embryonic murine hearts. To overcome these limitations, we took advantage of embryonic EPIcardial-derived Cells (EPICs), a continuous EPDC line established from embryonic murine epicardial cells (day 11.5 of development) that displays various EPDC properties, including differentiation potential into cardiovascular cell types, active cell migration, and ECM synthesis and proteolysis^50^. Please note that only one study has so far performed an analysis of embryonic epicardial-derived EVs. This study exclusively characterized the miRNA contents of epicardial-derived EVs but not EV protein content or epicardial-derived ECM composition^51^. Therefore, our results represent the first systematic analysis of the epicardial and EPDCs secretome, identifying potential roles for the epicardium in modulating cardiac homeostasis and responding to pathological stimuli.

## Materials and methods

### EPIC culture and extracellular vesicle isolation by ultracentrifugation

EPIC growth medium contains Dulbecco’s Modified Eagle’s Medium (DMEM, Gibco) supplemented with 10% Fetal Bovine Serum (FBS, Gibco), 100 U/mL penicillin (Sigma), 100 µg/mL streptomycin (Sigma), and 2 mM L-Glutamine (Sigma). For EVs isolation, a cell culture density of 2.7–3.8 × 10^3^ EPIC/cm^2^ was used (37 °C, 5% CO_2_). At 80% confluency, cells were cultured in EV-depleted medium (EPIC growth medium in which regular FBS is replaced by FBS ultracentrifuged at 100,000 g for 5.5 h at 4 °C, and filtered using a 0.2 µm-pore filter, UC-FBS). EPICs were then re-incubated at 37 °C, 5% CO_2,_ and either in 21% O_2_ atmosphere (“normoxic condition”, from here onwards EVs-N), 1% or 5% O_2_ (“hypoxic conditions”, EVs-H1% and EVs-H5%, respectively). EPIC-conditioned media was harvested every 24 h for two days, and then centrifuged at 10,000 g (4 °C, 50 min). The supernatant was ultracentrifuged again at 100,000 *g* (4 °C, 70 min). The pellet was then resuspended in 0.22 µm-filtered PBS (f-PBS). EVs were washed at 100,000 g (4 °C, 70 min). EPIC-derived EVs were finally resuspended in 100 µL of f-PBS for their use in further experimentation.

In this work, we have focused on two of the most studied types of EVs: small EVs, generally considered to present a size range of 30 to 200 nm, and medium/large EVs, with a size greater than 200 nm, following the criteria of the International Society of Extracellular Vesicles (ISEV)^37^.

### Transmission electron microscopy

Five microliters of each EVs suspension were loaded onto glow-discharged Formvar/Carbon-coated 200-mesh copper grids for 15 min at room temperature (RT). Adsorbed nanoparticles were negatively stained with 1% uranyl acetate for 30 s. Air-dried grids were observed using a Tecnai G2 20 Twin microscope (Thermo Fisher Scientific) operating at 120 kV. Three biological replicates were observed under TEM for EVs-N and EVs-H5%. For EVs-H1%, two biological replicates were analyzed.

### Nanoparticle tracking analysis

EVs particle concentration was measured with Nanosight NS300 (Malvern Instruments, UK) and Nanoparticle Tracking Analysis (NTA) software version 3.2 (Malvern Instruments). EVs were diluted at 1:1000 and 1:2000 in sterile water and injected into the flow cell. Samples were analyzed at identical flow rates, camera settings, and analysis detection thresholds. To confirm the cleanliness of the flow cell, measurements were also performed with sterile water. Four consecutive acquisitions (30 s, 25 frames/second, 25ºC) were recorded per sample (n = 3).

### Extracellular matrix isolation

To isolate EPIC-derived extracellular matrix (EPIC-ECM), 5.3 × 10^3^ EPIC/cm^2^ were seeded in EPIC growth media that was refreshed daily until full confluency was reached. At this point, EPICs were kept in culture for three additional days to obtain an enriched ECM deposition^52,53^. NH_4_OH (20 mM) was used to induce decellularization by an osmotic shock^54^. Soluble (EPIC SM) and insoluble (EPIC IM) phases were retrieved. For functional assays, urea (2 M) dissolved in cold sterile PBS and filtered using 0.22-µm filter, was added to the EPIC IM fraction still attached to the plate; then the samples were scraped on ice. Plates were incubated at 4 °C for 72 h in a rocking shaker. EPIC IM has scraped again and washed with cold sterile PBS and concentrated (250 µL) using Amicon Ultra 3 kDa cutoff centrifugal filter units (Amicon).

### Animal experimentation and embryonic heart decellularization

Animals used in this study were handled in compliance with institutional and European Union guidelines for animal care and welfare under a specific experimental procedure approved by the Ethics Committee of the University of Málaga. In addition, we confirm that all experiments were performed in accordance with ARRIVE guidelines. Mouse embryos were staged based on the presence of a vaginal plug as embryonic day 0.5 (E0.5). CD1 pregnant females were anesthetized with isoflurane in an induction chamber, sacrificed by cervical dislocation, and then embryos were isolated from the uterus and washed in PBS. Six E17.5 embryonic hearts were excised, washed with PBS, and decellularized. Briefly, the hearts were incubated at room temperature for 4.5 h in 20 mM NH_4_OH. The resulting embryonic cardiac mesh was then washed four times in MilliQ water and homogenized in SDS-PAGE buffer [6% (v/v) glycerol, 2% (m/v) SDS, 100 mM dithiothreitol (DTT) and 0.05 M Tris–HCl pH 6.8]. Samples were stored at − 80 °C before LC–MS/MS analysis.

### Proliferation assay

To evaluate the effect of EPIC-derived EVs on either EPIC or human umbilical vein endothelial cell (HUVEC) proliferation, 20 or 50 µg/mL of labeled EVs-N, EVs-H5%, or EVs-H1% were diluted in specific starving media added to the cells at 70% confluence and incubated for 24 h (n = 3). These starving media contained 0% FBS for EPIC and 1% FBS for HUVECs (see Supplementary Material for additional information). To evaluate the effect of two different EPIC-derived ECM fractions on HUVEC proliferation, cells (n = 3) were seeded at 1.3–1.4 × 10^4^ cells/cm^2^ in complete medium (EGM-2) and incubated for 3 or 5 days at 37 °C and 5% CO_2_ in culture plates coated with different formulations: (i) 1% gelatine (negative control), (ii) 1% gelatine containing 100 µg EPIC IM, (iii) 1% gelatine containing 100 µg EPIC SM, (iv) 1% gelatine containing 100 µg Matrigel®(Corning), (v) 1% gelatine containing 100 µg EPIC IM plus 100 µg Matrigel®, and (vi) 1% gelatine containing 100 µg EPIC IM plus 100 µg EPIC SM plus 100 µg Matrigel®. Coated plates were incubated for 2 h at 37 °C in the coating solution. To quantify cell numbers in the experiments including EPIC-derived EVs or cells plated on EPIC IM- and EPIC SM-coated plastic dishes, incubations were carried out in the presence of 5-Ethynyl-2´-deoxyuridine (EdU) (further details in the supplemental material file).

### Proteomic analysis of EPIC-derived secretome

For a quantitative analysis of the EPIC-derived EVs, Tandem Mass Tag (TMT) proteomic analysis was employed (n = 3 per condition). Proteins were diluted to 1 µg/µL and extracted using Tris (2-carboxyethyl) phosphine (TCEP) and tetraethylammonium bromide (TEAB) buffers and finally digested with trypsin. Proteins were tagged using a TMT10plex Isobaric Mass Tagging Kit (Thermo Fisher Scientific) according to the manufacturer’s instructions. The tagged EVs were analyzed using Liquid Chromatography with tandem mass spectrometry (LC–MS/MS).

For a qualitative analysis of ECM composition, label-free bottom-up mass spectrometry was used. EPIC IM (n = 3), EPIC SM (n = 3), decellularized E17.5 hearts (n = 6), and Matrigel® (n = 3) were denatured and purified for LC–MS/MS analysis. Briefly, purified samples were injected into an Easy nLC 1200 UHPLC coupled to Q Exactive HF-X Hybrid Quadrupole-Orbitrap mass spectrometer (ThermoFisher Scientific). Peptides were separated at a 20 μL/min flow rate in a 50 cm analytical column (PepMap RSLC C18, 2 µm, 100 A, 75 µm × 50 cm; Thermo Fisher Scientific). For both ECM and EVs peptides, elution was performed at a constant flow of 300 nL/min. The resolution of MS survey scans was set to 120,000 m/z, while for MS/MS the resolution was set to 30,000. The ion pulverization voltage was set to 2.2 kV with an m/z window from 350 to 1500, an isolation window of 0.7 m/z, and a dynamic exclusion of 20 s. Software versions used for data acquisition and manipulation were Tune 2.9 and Xcalibur 4.1.31.9.

### Bioinformatics analysis and statistics

For all EVs and ECM studies, a minimum of three biological replicates were assessed per condition. One-way or two-way ANOVA tests were performed using GraphPad software version 8.0.2. A *p*-value < 0.05 was considered statistically significant. For TMT10plex data, the *MaxQuant* (Version 1.5.4.1) software was used to identify proteins from the RAW files together with the *SwissProt* mouse protein database (release 2019_11) supplemented with common contaminants^55,56^. Proteins that were not identified in at least two samples of each experimental condition were not considered for statistical analysis. Multivariate statistical analyses (principal component analysis–PCA- and agglomerative hierarchical cluster analysis) were performed using R/Bioconductor^57^. For statistical analysis, a limma test was employed in which proteins with a *p*-value < 0.05 were considered differentially expressed^58^. Then, EVs proteins found to be accumulated were associated with Gene Ontology (GO) terms using *clusters_to _enrichments.*R script from ExpHunterSuite^59,60^. For label-free LC–MS/MS data, *Sequest HT* was used. Exploratory protein identification was pursued by identifying protein presence or absence in two or more biological samples with three replicates for EPIC IM, EPIC SM and Matrigel® and six replicates for E17.5 decellularized hearts. Proteins were considered part of an experimental condition if they were present in at least two replicates. The false discovery rate (FDR) for consecutive protein and peptide assignments was determined using the *Percolator* software package. GO analysis was performed at a qualitative level (presence or absence). Lists of proteins were also associated with GO terms using *clusters_to _enrichments*.R script. This script uses over-representation analysis (ORA) that selects a group of significant proteins and performs a Fisher’s exact test for each GO term. Fisher’s exact test *p*-value were corrected with the Benjamini–Hochberg procedure^60^, the latest version of the code can be found at https://github.com/seoanezonjic/ExpHunterSuite). GO terms with adjusted *p* value higher than 0.05 were discarded.

## Results

### Hypoxia affects the heterogeneity of epicardial-derived extracellular vesicles

**Fig. 1:**
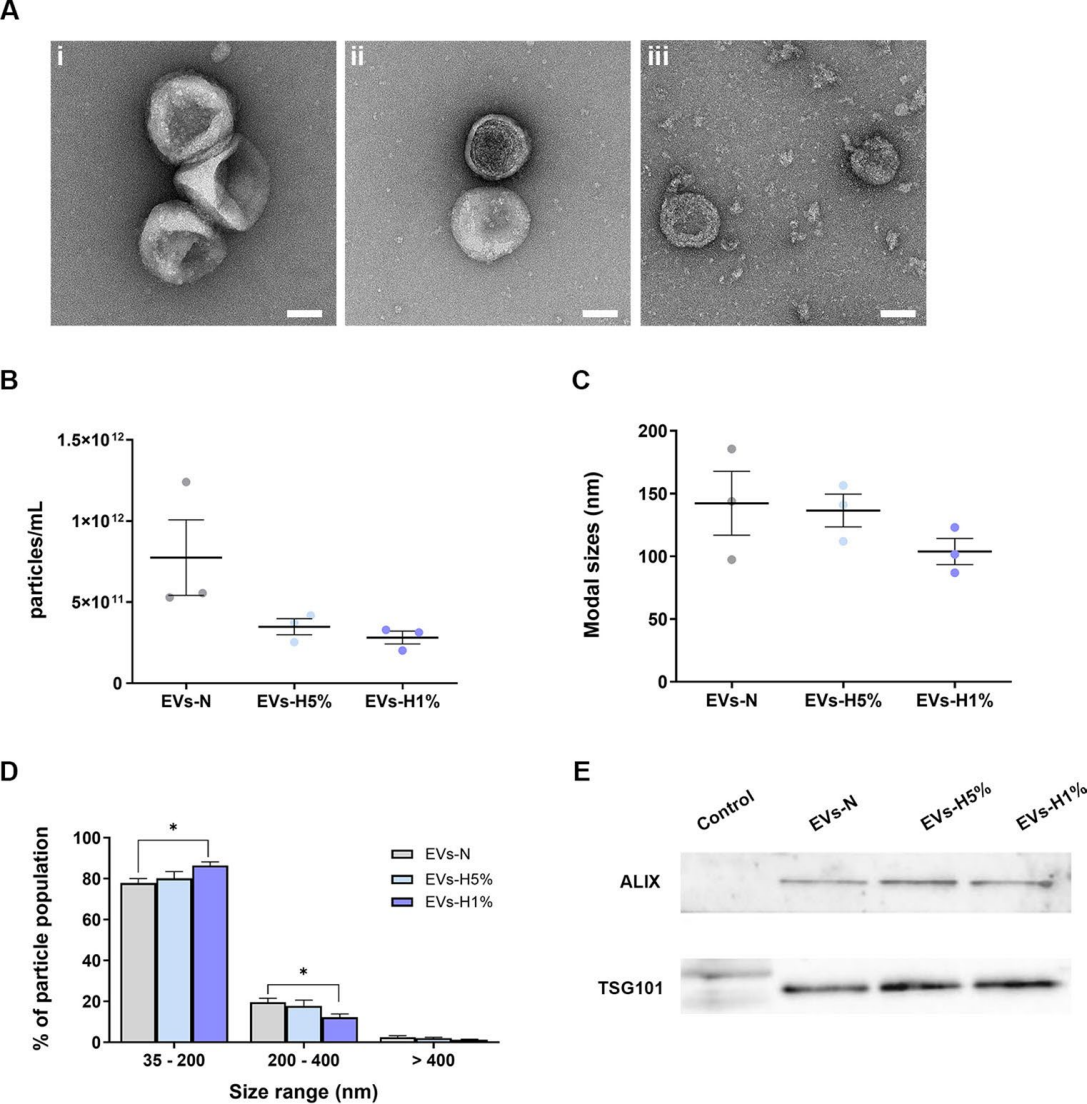
Characterization of EPIC-derived EVs. (**A**) Representative TEM images of EVs-N (i), EVs-H5% (ii), and EVs-H1% (iii) samples. Scale bar: 50 nm; (**B**) Particle concentration (number of particles/mL) of EVs-N (7.7 ± 2.3 × 10^11^ particles/mL), EVs-H5% (3.5 ± 0.5 × 10^11^ particles/mL) and EVs-H1% (2.8 ± 0.4 × 10^11^ particles/mL); (**C**) Modal sizes of EVs-N (142.2 ± 25.5 nm), EVs-H5% (136.5 ± 13.1 nm) and EVs-H1% (103.8 ± 10.5 nm); (**D**) Distribution of particle population in three different size ranges from EVs-N (35–200 nm: 73.8 ± 2.2%; 200–400 nm: 19.6 ± 1.9%; > 400 nm: 2.5 ± 0.6%), EVs-H5% (35–200 nm: 80.1 ± 3.3%; 200–400 nm: 17.8 ± 1.9%; > 400 nm: 2.1 ± 0.4%) and EVs-H1% (35–200 nm: 86.4 ± 1.7%; 200–400 nm: 12.3 ± 1.6%; > 400 nm: 1.3 ± 0.3%); (**E**) Representative images of western blot against ALIX (95 kDa) and TSG101 (45 kDa) accumulation in protein lysates from total mouse embryos at E11.5 (control), EVs-N, EVs-H5%, EVs-H1%. Data are presented as mean ± SEM, n = 3. One-way ANOVA test for particle concentration and modal size. Two-way ANOVA test for particle size distribution, *p < 0.05.

The epicardium and subepicardium are low oxygen tension environments. Because of that, we have compared EVs isolated from EPICs cultured under different oxygen levels: 21%—normoxia (EVs-N), 5% (EVs-H5%) or 1% (EVs-H1%)—hypoxia (Table 1). Remarkably, no changes in viability or growth rate of EPIC were observed between experimental conditions. First, Transmission Electron Microscopy (TEM) analysis did not reveal significant structural differences between EVs-N, EVs-H5% and EVs-H1% (Fig. 1A). EVs showed a trend to equally collapse in all samples, as previously described for dehydrated EVs^61^.

**Table 1:** Summary of EVs-N, EVs-H5%and EVs-H1% characterization and proteomic enrichment.

	EVs-N	EVs-H5%	EVs-H1%
Morphology (TEM)	Cup-shaped structure	Cup-shaped structure	Cup-shaped structure
Particles < 200 nm	73.8 ± 2.2%	80.1 ± 3.3%	86.4 ± 1.7%
EV concentration (particle/mL)	7.7 ± 2.3 × 10^11^	3.5 ± 0.5 × 10^11^	2.8 ± 0.4 × 10^11^
EV-related proteins (ALIX and TSG101)	Present	Present	Present
Glycolytic protein enrichment (fold change)	1	1.04–1.2	1.47–1.95
Most enriched glycolytic enzymes	-	TPI1, PGK1, LDHA, PKM, GAPDH, ENO1, PGAM1	TPI1, PGK1, LDHA, PKM, GAPDH, ENO1, PGAM1

*NE = Non-enriched.

Next, we compared EVs size and concentration using Nanoparticle Tracking Analysis (NTA) (Fig. 1B–D). EVs-N, EVs-H5%, and EVs-H1% showed no difference either in particle concentration (Fig. 1B) or modal particle size (Fig. 1C) as oxygen concentration was reduced. We then analyzed the distribution of the particles in three size categories, i.e. small (35–200 nm), medium (200–400 nm), and large (> 400 nm) for each condition. EVs-H1% samples showed a significant increase in the proportion of small EVs and a significant decrease in medium sized EVs (as compared to EVs-N) (Fig. 1D). Small EVs enrichment was confirmed by western blot analysis, detecting proteins typically associated with this type of EVs, such as ALIX and TSG101 (Fig. 1E). Sample loading accuracy was assessed by Ponceau Red staining (Fig. S1).

### Hypoxic epicardial-derived extracellular vesicles are enriched in glycolysis/glucogenesis-related proteins

**Fig. 2:**
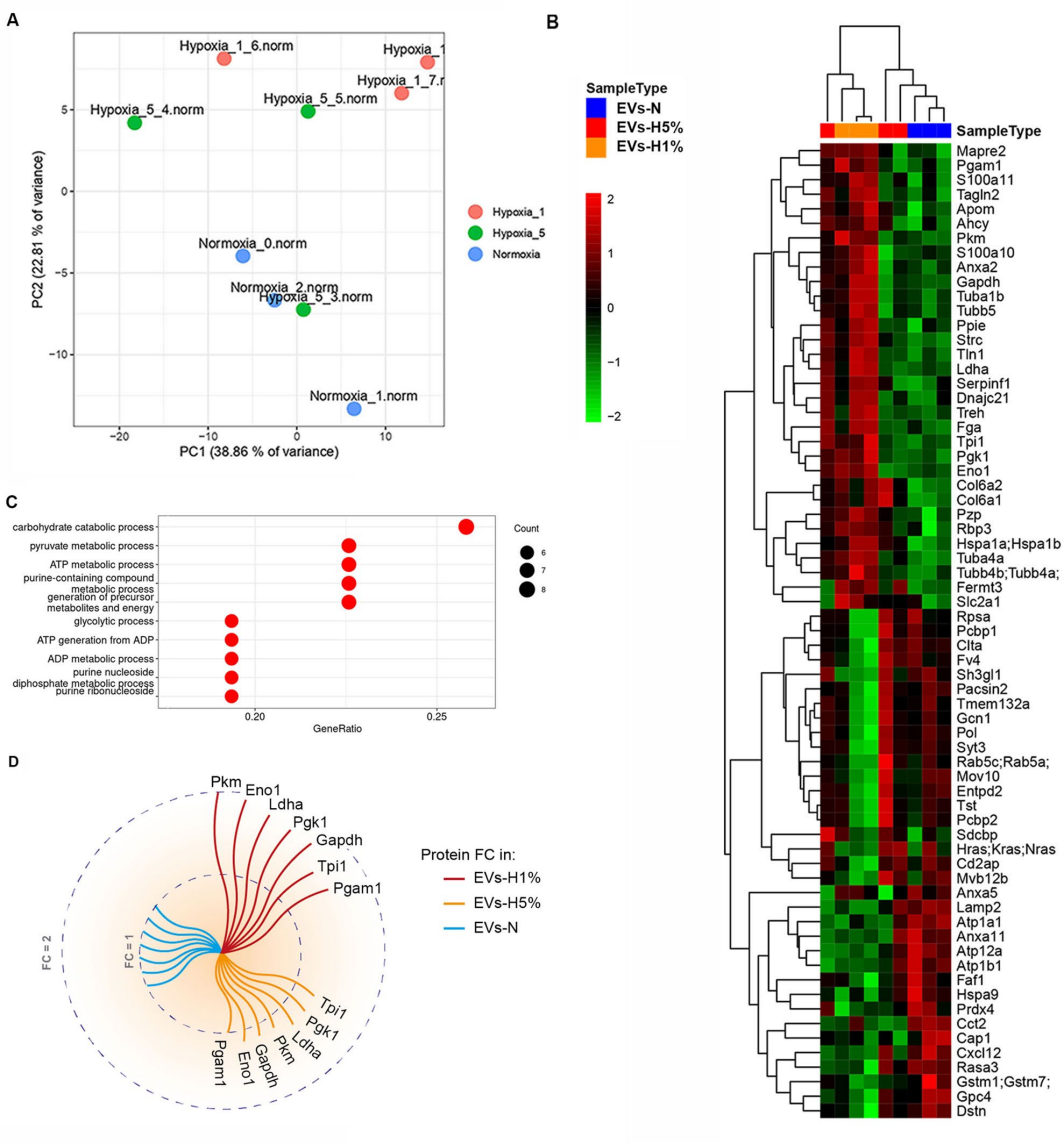
Proteomic analysis of epicardial-derived EVs isolated from EPIC cultured in normoxic or hypoxic conditions. (**A**) PCA representation shows identified proteins from EVs-N (“Normoxia_0.norm”, “Normoxia_1.norm” and “Normoxia_2.norm”), EVs-H5%, (“Hypoxia_5_3.norm”, “Hypoxia_5_4.norm” and “Hypoxia_5_5.norm”), and EVs-H1% (“Hypoxia_1_6.norm”, “Hypoxia_1_7.norm” and “Hypoxia_1_8.norm”); (**B**) Heatmap representation of identified proteins that significantly differ between EVs-H5% or EVs-H1% and EVs-N (*p*-value < 0.05; n = 3). Scale represents standardized log_2_ of the protein abundancies with a mean of zero and standard deviation of one; (**C**) Ten most representative GO annotations of biological process from significantly up-regulated proteins in EVs-H1% as compared to EVs-N. The x-axis represents the protein ratio, dot size corresponds to the number of proteins associated with the functional category, and dot colour corresponds to the adjusted *p* value < 0.05; (**D**) Graphical representation of the fold change increase of glycolysis-related protein abundancy in EVs-H5% and EVs-H1% in respect to EVs-N. The inner circle corresponds to a fold change (FC) of 1 and the outer circle to an FC of 2.

To characterize the effect of hypoxia in EPIC-derived EVs, we performed a TMT10plex proteomic study. A principal component analysis (PCA) representation identified that EVs-N and EVs-H1% proteomes differ each other, whereas the EVs-H5% showed an overlap between both sample groups (Fig. 2A). Next, we built a hierarchical clustered heatmap that showed significant differences between EVs-H5% or EVs-H1% and EVs-N (adjusted *p*-value < 0.05) (Fig. 2B). This heatmap revealed that: (1) 31 proteins were overrepresented in EVs-H1% as compared with EVs-N; (2) another 15 were overrepresented in both EVs-N and EVs- H5% relative to EVs-H1%; and (3) 20 proteins were reduced as oxygen levels in EPIC cultures decrease.

Then, we performed a Gene Ontology (GO) analysis of the up-regulated proteins present in EVs-H1% in comparison with EVs-N (pvalue < 0.05) (Fig. 2C). Interestingly, glycolysis-related biological processes, such as carbohydrate catabolic, pyruvate or glycolytic processes, were identified among the most significant terms (adjusted *p* values < 1.09 × 10^−9^). Other GOs related to the analyzed proteins were associated with nucleoside and nucleotide phosphorylation, processes commonly related to energy production or to nucleic acid synthesis or degradation, but with a lower *p*-value that the ones related to glycolysis or other related processes (data not shown). In accordance with these data, seven glycolytic metabolism-related enzymes showed an increase in their abundancy in EVs-H1% as compared to EVs-N: phosphoglycerate mutase 1 (PGAM1), pyruvate kinase muscle isozyme (PKM), glyceraldehyde-3-phosphate dehydrogenase (GAPDH), L-lactate dehydrogenase A chain (LDHA), triosephosphate isomerase (TPI1), phosphoglycerate kinase 1 (PGK1) and α-enolase (ENO1)(fold change between 1.47 and 1.95) (Fig. 2D). When analyzing the fold change of the same set of proteins from EVs-H5% extracts, the FC related to EVs-N ranges from 1.04 to 1.2 (Fig. 2D). Altogether, these data suggest that the protein composition of EPDC-derived EVs is remarkably sensitive to the oxygen available in their milieu, so that EVs showed a significant and progressive enrichment in glycolytic enzymes when hypoxia is increased in parenteral cell cultures.

### Extracellular vesicles derived from hypoxic epicardial-derived cells exert both autocrine and paracrine effects in surrounding cells

Based on the results from TMT10plex analysis, we hypothesized that epicardial-derived cells could affect the glycolytic function of surrounding cells via EVs. To assess this, we studied the potential autocrine effect of EPIC-EVs on the EPIC line itself. In parallel, the paracrine effect of these EVs was also tested on human umbilical cord-derived endothelial cells (HUVECs).

**Fig. 3:**
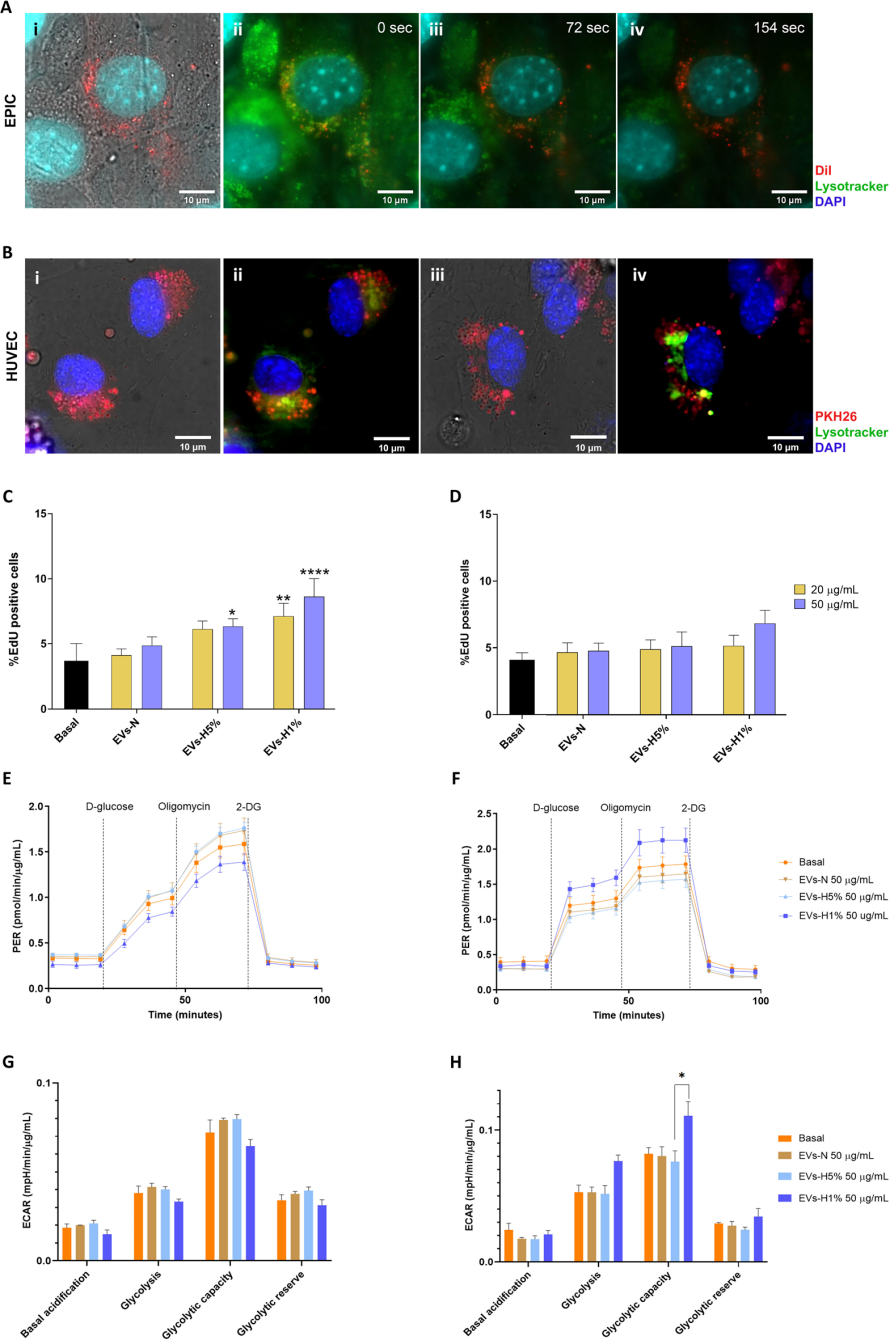
Autocrine and paracrine effect of the cargo of epicardial-derived EVs. (**A**) Representative image of EPICs after incubation with EVs stained with DiI (EVs-DiI). (i) Bright-field image of EPIC nucleus stained with DAPI containing EVs-DiI (red); frames of time-lapse of EPIC stained with Lysotracker (acidic organelles in green) after the incubation with EVs-DiI at time 0 s (ii), 72 s(iii) and 154 s (iv); (**B**) Representative images of HUVECs incubated with PKH26-stained EVs. (i) and (iii) show bright field images of HUVECs nucleus stained with DAPI containing PKH26-stained EVs (red); (ii) and (iv) show HUVECs containing PKH26-stained EVs, acidic organelles stained with Lysotracker (green) and nuclei stained with DAPI. Scale bars: 10 μm. (**C**) Percentage of EdU^+^ EPIC incubated for 24 h at basal condition (grey bar). 20 μg/mL (yellow bars) or 50 μg/mL (blue bars) of EVs-N, EVs-H5% or EVs-H1% (*p < 0.05, **p < 0.01, ****p < 0.0001 against basal condition); (**D**) Percentage of EdU^+^ HUVECs incubated for 24 h at basal condition (black bar). 20 μg/mL (yellow bars) or 50 μg/mL (blue bars) of EVs-N, EVs-H5% or EVs-H1%; (**E**) PER profiles of EPIC or (**F**) HUVECs incubated with 50 µg/mL of EVs-N, EVs-H5% or EVs-H1% or at basal condition (n = 3); (**G**) Glycolysis stress test parameters (basal acidification, glycolysis, glycolytic capacity, and glycolytic reserve) were calculated for EPIC and (**H**) HUVECs incubated with 50 µg/mL of EVs-N, EVs-H5% or EVs-H1% or at basal condition (n = 3).

First, to evaluate the internalization of EPIC-EVs, samples were stained with lipophilic dyes just before incubating them with different cell types. On one hand, EVs-N were stained with DiI (EVs-DiI; Fig. S2) and added to cultured EPICs. The internalization of EPIC EVs was confirmed by the counterstaining of acidic organelles of EPIC with LysoTracker™ (Fig. 3A). On the other hand, EPIC-EVs internalization by HUVECs was also confirmed after staining EVs (Fig. 3B). Second, to evaluate potential changes in the biology of target cells, cell proliferation was analyzed in both EPIC and HUVECs after incubation with different concentrations (20 or 50 ug/mL) of each type of EVs (Fig. 3C–D). No significant differences in cell proliferation were found between the basal condition and EPIC incubated with EVs-N or 20 ug/mL of EVs-H5%. However, the number of EdU^+^ EPIC was significantly increased after incubation with 50 ug/mL EVs-H5%, and both concentrations of EVs-H1% (Fig. 3C). On the other hand, HUVECs proliferation did not significantly change after incubation with each EVs type as compared to its basal condition (Fig. 3D). Interestingly, no signs of cytotoxicity or changes in cell morphology were observed in either EPIC or HUVECs under any of these conditions.

Next, we evaluated the capacity of EPIC-derived EVs as potential metabolic modulators. In order to do so, the glycolytic response of EPIC or HUVECs was analyzed using a Seahorse Glycolysis Stress Test after their incubation with 50 μg/mL of EVs-N, EVs-H5%, or EVs-H1%.

None of the different populations of EVs induced significant changes to the glycolytic behavior of EPIC (Fig. 3E and G). However, HUVECs incubated with EVs-H1%, HUVECs drastically increased their glycolytic capacity in comparison with HUVECs incubated with EVs-H5% (Fig. 3F and H). This data suggests that EPIC-EVs can modify the metabolism of the receiving cells.

### Soluble and insoluble protein fractions can be isolated from epicardial-secreted extracellular matrix

**Fig. 4:**
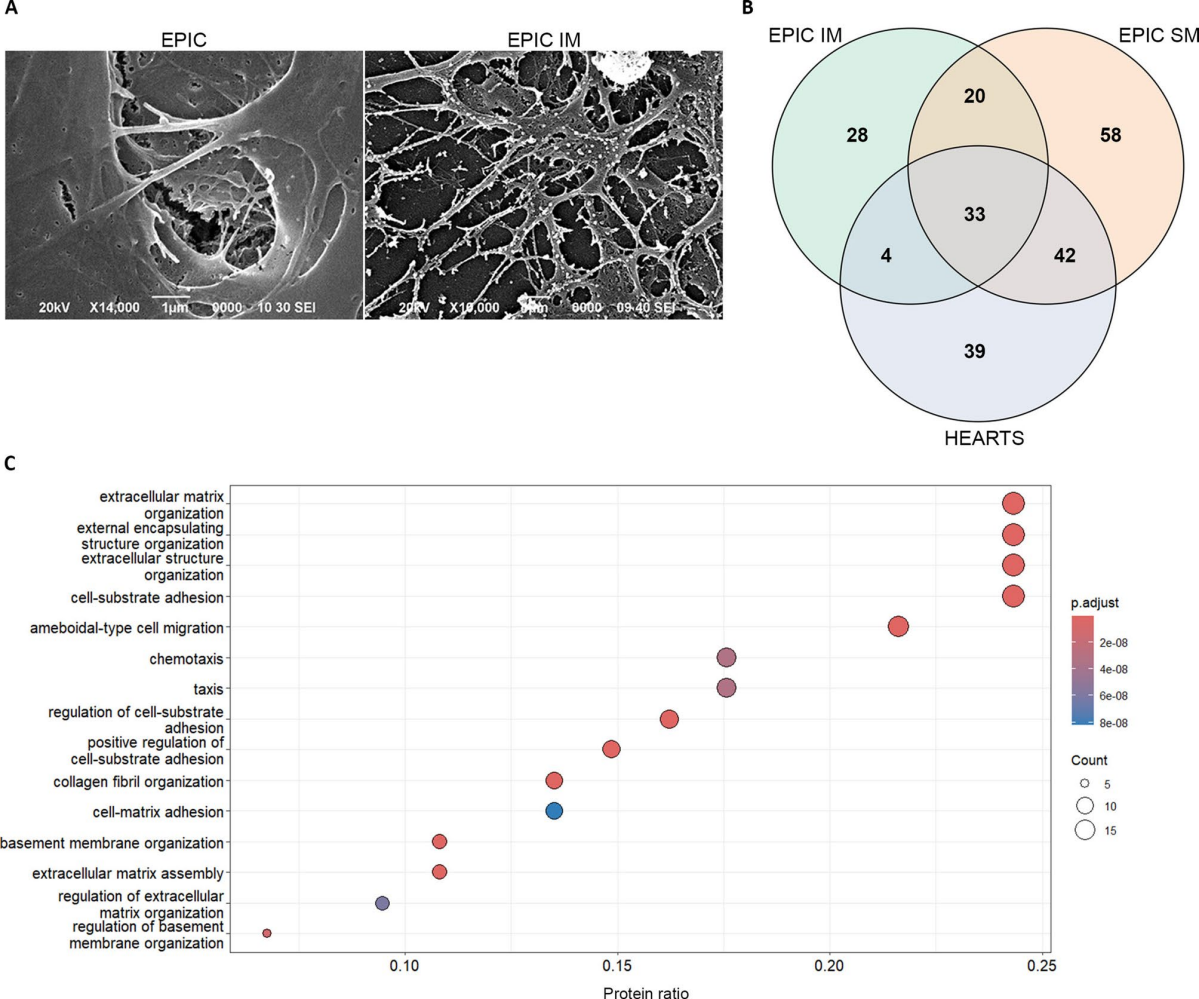
EPIC-derived ECM characterization and function assessment. (**A**) representative SEM images of EPIC and EPIC-derived ECM at 14,000X and 10,000X magnification, respectively; (**B**) Venn diagram of extracellular proteins identified in EPIC IM, EPIC SM, and E17.5 hearts; (**C**) Biological processes (GO analysis) of extracellular proteins in common between both EPIC-ECM fractions and E17.5 hearts/DMEH. The most highly significant categories in descending order in categories of gene ratio, are defined as the proportion of significant genes that are found in the functional category. The X-axis represents the gene ratio and the dot size the number of genes associated with the functional category and the dot colour corresponds to the adjusted *p* value < 0.05.

To analyze another potentially important element of the epicardial secretome, we explored the composition of the epicardial-derived extracellular matrix (EPIC ECM). The experimental procedure used to obtain the EPIC -ECM directly resulted in the differential isolation of two ECM fractions, a soluble matrix component (EPIC SM) and an insoluble one (EPIC IM). For this reason, we decided to characterize both fractions separately. When analyzed by scanning electron microscopy, the EPIC IM fraction displayed a rough surface and a clear irregular organization in comparison with EPIC, that showed fibers assembled to form a mesh-like organization (Fig. 4A). Routine immunohistochemical analysis of both EPIC- ECM fractions revealed they are enriched in both basement membrane and ECM proteins and contained low (EPIC SM) or negligible (EPIC IM) amounts of intracellular proteins (Fig. S3).

To determine their extracellular protein composition, both ECM fractions were submitted to shotgun proteomics. Based on GO annotations, 24% of EPIC IM and 9% of EPIC SM proteins were identified as “extracellular”. A total of 53 extracellular proteins were found to be common between the two fractions. This includes several cardiac-enriched ECM proteins such as hyaluronan, proteoglycans, elastin, fibrillin, and tenascin (Table S1). As expected, these proteins are associated with biological processes related to the ECM such as extracellular matrix organization, extracellular structure organization (wound healing or cell-substrate adhesion among others) (adjust *p*-value < 1 × 10^−7^) (Fig. S4).

Then, we identified the subset of proteins specifically present in each EPIC-ECM fraction. For EPIC IM, 32 unique proteins were identified, including more than ten types of collagen and other proteins related to cardiac ECM such as agrin (AGRN) or osteonectin (ON), and growth factors related proteins such as fibroblast growth factor receptor 2 (FGFR2), platelet-derived growth factor (PDGF) or pigment epithelium-derived factor (SERPINF1) (Table S2). In the case of EPIC SM, 100 unique proteins were found, most of them related to classical signaling pathways involved in cardiac ECM development and repair, such as stromal cell-derived factor 1 (CXCL12), platelet-derived growth factor subunit B (PDGFB), transforming growth factor β-2 proprotein (TGFB2), or Wnt-7a (WNT7A) (Table S2). The protein signature of the two fractions can be associated with similar GO terms related to angiogenesis, migration, proliferation, or cell adhesion (Fig. S5). However, EPIC IM proteins showed a stronger association with these GO terms than the ones from the EPIC SM fraction (please, compare X- axis between both graphs in Fig. S5).

### Comparative analysis of the epicardial-derived extracellular matrix composition

To evaluate the potential significance of the epicardial contribution to cardiac ECM, we compared both EPIC-derived ECM fractions with the ECM isolated from a pool of decellularized murine embryonic hearts (DMEH). Shotgun proteomics identified 33 extracellular proteins in common between the three datasets, such as fibronectin (FN-1), collagen α-1(I) chain (COL1A1), or collagen α-2(I) chain (COL1A2) (Fig. 4B, Table S3). In addition, EPIC SM and DMEH exclusively share 42 proteins including different types of collagens including α-1(IV) (COL4A1) and α-2(IV) (COL4A2), α-1(VI) (COL6A1) and α-2(VI) (COL6A2) (Fig. 4B). EPIC IM shows 4 common proteins with DMEH and were not found in EPIC SM (Table S3). To better characterize the similarities between EPIC ECM and DMEH, a GO term analysis was performed for the 79 proteins that both fractions of EPIC ECM have in common with DMEH (Fig. 4C). As expected, these proteins were associated with biological processes related to ECM organization, wound healing, cellular adhesion and migration, and molecular signaling. In addition, 67% of the extracellular protein detected from EPIC ECM are found in DMEH, showing a high degree of similarity between EPIC ECM and DMEH.

To inquire about the biological properties of EPIC-derived ECM based on its protein composition, we compared both EPIC ECM datasets with Matrigel®, a widely used commercial ECM for in vitro cell growth studies. Using label-free LC–MS/MS, 314 proteins were identified in Matrigel® samples, but only 89 of them were annotated as “extracellular”. Out of these 89 extracellular proteins, 46% were found to be exclusive for Matrigel® and associated with GO terms related to the negative regulation of different enzymatic activities, such as hydrolase, peptidase or proteolysis (Fig. S6A, B). In contrast, 23 extracellular proteins were shared between Matrigel®, EPIC IM and EPIC SM samples (Fig. S6A). Most of these common proteins belong to laminin (LAM) and heat shot protein (HSP) families and are associated with GO terms related to extracellular functions, such as extracellular matrix organization or substrate adhesion-dependent cell spreading (Fig. S6C). Taken together, our results indicate that the proteins found in the EPIC-ECM fractions are substantially different from those identified in Matrigel®.

To further evaluate the specificity of EPIC IM and SM fractions, all our generated datasets (EPIC IM, EPIC SM, DMEH, Matrigel®) were compared (Table 2). The first analysis we carried out revealed that only 17 extracellular proteins, associated with LAM and HSP families, were shared between the four datasets (Fig. S7A and Table S4). A GO term analysis from these 17 proteins identified a series of biological processes related to cell attachment, flattening, and differentiation (Fig. S7B). To unravel the specificity of the composition of each sample, the relative abundances of extracellular proteins in the total protein pool per condition were calculated (Table S5). Fibronectin (FN-1) and other extracellular proteins associated with FN, such as serine protease HTRA1, were highly enriched in EPIC ECM fractions. In contrast, Matrigel® was enriched in proteins associated with the basement membrane structure, such as the ones of the family of laminin (LAMA1, LAMB1 and LAMC1) (Table 2). These results were validated by western blot (Fig. S8) and clearly indicate that EPIC ECM contains an important number of proteins secreted to the extracellular milieu. This contrasts with Matrigel®, whose composition is closer to that of a basement membrane.

**Table 2:** Summary of the proteomic comparison between the four datasets generated in this study (EPIC IM, EPIC SM, DMEH, and Matrigel®).

	EPIC IM	EPIC SM	DMEH	Matrigel®
GO terms of biological processes	Epithelial cell proliferation			Cell attachment, flattening and differentiation
	ECM organization Response to wounding/wound healing Cell substrate adhesion			
Proteins in common	53			
	33			
	17			
Enriched proteins	EMILIN-1 HTRA1 CXCL12	ANXA2 HSP90B1	COL1A2	LAMB1 LAMC1 NID-1
	FN1			
		MYH9 HSPA5		
			HSPG	
	LAMA1			LAMA1

### Epicardial-derived extracellular matrix induces the proliferation of endothelial cells

**Fig. 5:**
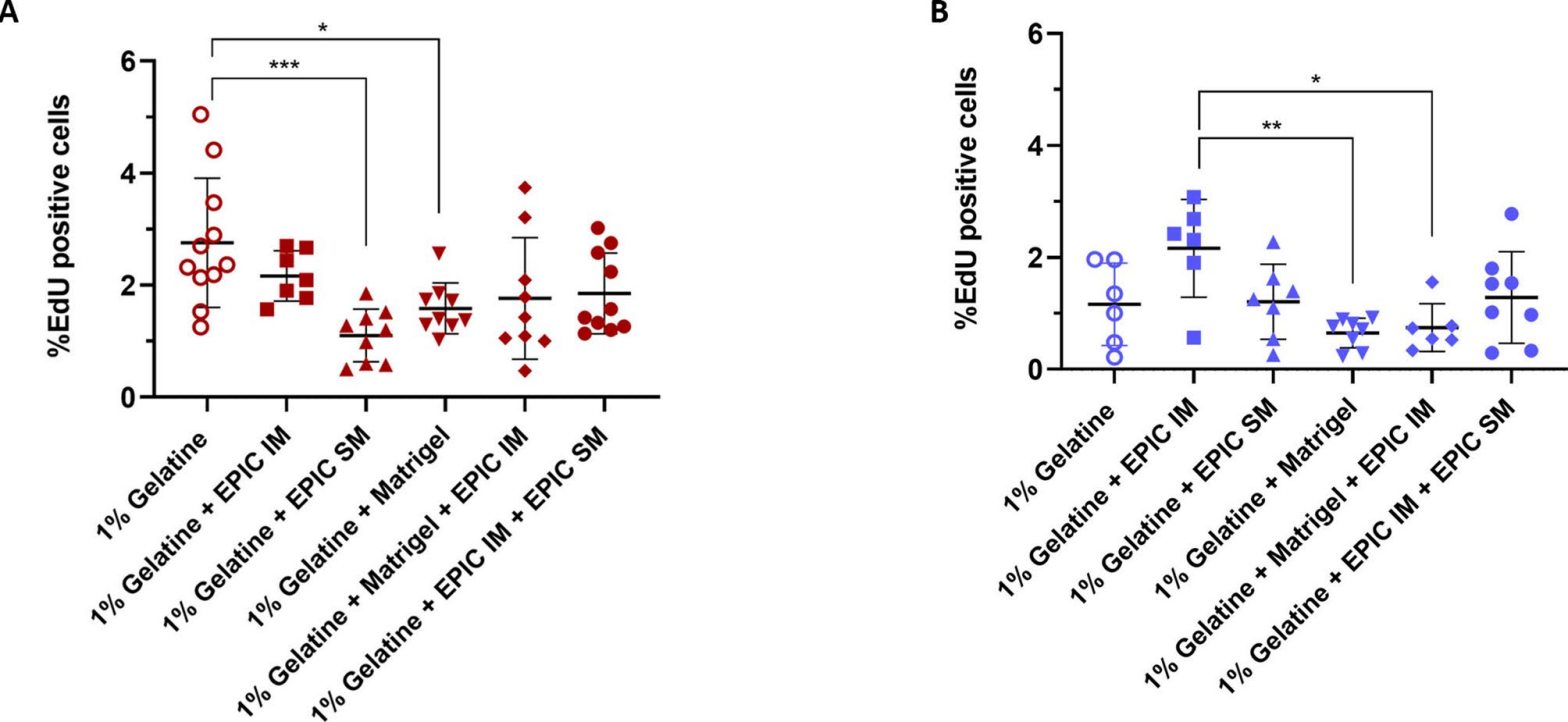
EPIC-derived ECM function assessment. HUVECs were incubated with 1% gelatine, 1% gelatine + 100 μg/mL of EPIC IM, 1% gelatine + 100 μg/mL of EPIC SM, 1% gelatine + 100 μg/mL Matrigel, 1% gelatine + 100 μg/mL Matrigel + 100 μg/mL of EPIC IM, or 1% gelatine + 100 μg/mL of EPIC IM + 100 μg/mL of EPIC SM for three and five days. (**A**) Percentage of EdU positive HUVECs after incubation for three days; (**B**) Percentage of EdU positive HUVECs after incubation for five days; *p < 0.05, **p < 0.01, ***p < 0.001, ****p < 0.0001.

To validate whether the differences in protein composition involve changes in their biological properties, we performed a functional study to evaluate the potential effect of EPIC-ECM on HUVECs growth. HUVECs were cultured on wells coated with different substrates, combining gelatine with EPIC IM, EPIC SM, and/or Matrigel® (Fig. 5). The HUVECs proliferative rate, measured as a percentage of EdU-positive cells, was evaluated after three (Fig. 5A) and five days (Fig. 5B) of culture.

After three days, the proliferation rate of HUVECs cultured on gelatine plus EPIC SM or Matrigel® was significantly lower than in wells coated only with gelatine (Fig. 5A). However, after five days, HUVECs plated onto Matrigel®, with or without EPIC IM, showed a lower percentage of EdU-positive cells versus cells cultured on the EPIC IM fraction alone (Fig. 5B). These results indicate that EPIC IM and Matrigel® have functionally different effects on HUVECs growth during the 5-day assay.

## Discussion

Several reports have demonstrated the importance of the epicardium and epicardial-derived mesenchymal cells (EPDCs) during the embryonic formation, growth, and maturation of the heart. As pioneers in the colonization of the nascent cardiac interstitium, EPDCs actively participate in the conditioning of the extracellular cardiac microenvironment through the secretion of multiple molecules into the extracellular milieu, both during normal embryonic development and disease^62,63^. However, despite its enormous relevance, there have been few studies into the epicardial/EPDC-derived secretome. This is due to technical challenges mostly related to the low number of native epicardial and EPDCs that can be retrieved from cardiac explants for research purposes. To circumvent this limitation, we took advantage of EPIC, an immortal cell line derived from mouse embryonic epicardium that has been shown to recapitulate an important part of embryonic epicardial biology^50^. As we are aware that this in vitro cellular model is unlikely to fully recapitulate the biology of native epicardial cells, we have, when possible, compared results from EPIC with other in vivo data.

In this work, two fractions of the epicardial secretome were studied: EVs and ECM. EVs are relevant to cell-to-cell communication^64–66^. Since the embryonic heart develops under low oxygen tension conditions^31,67^, and hypoxia is known to modulate the differentiation of EPDCs^27,29,30,32^, the effect of hypoxia over epicardial-derived EVs was investigated. Although EPIC-derived EVs were found to be morphologically similar to those released by other cell types^68,69^, we discovered that hypoxic EPICs secreted smaller-sized EVs than normoxic ones, as reported for other cell types in different biological contexts^70–73^. Moreover, as oxygen levels in EPIC cultures decrease, the protein signature of EPIC-derived EVs gradually shifts towards a composition enriched in glycolysis/gluconeogenesis enzymes. Interestingly, this enrichment was accentuated in EVs-H1% versus EVs-H5%, indicating that the cargo of EPDC-derived EVs is remarkably sensitive to the oxygen available in their milieu.

Our study also shows that EVs isolated from EPIC cultured under hypoxia were able to enhance EPIC proliferation in an autocrine manner. These results complement data from previous works showing that EVs from hypoxic cell cultures are able to promote proliferation in different cells^74,75^. Such EVs were found to be enriched in proteins like PKM2 and PGAM1, which were also found in our hypoxic EVs. This suggests that EPIC EVs act in both a paracrine and an autocrine manner. Interestingly, PKM2, PGAM1 or their isoforms (e.g. phosphoglycerate mutase 2/PGAM2) affect the cell metabolism at different levels, including the mitochondrial respiration rate and the generation of reactive oxygen species^76^. Based on these evidences, we consider two plausible scenarios: 1) epicardial cells may have the capacity to respond to high energy demands without drastically changing their metabolism, as a pre-adaptation to their cardiac hypoxic niche during development^31^; or 2) this proliferative effect could result from the immortalization process of EPIC, which could have switched the metabolism of these cells into one more dependent on amino acids such as L-Glutamine than on glucose, as described in other immortalized cell lines^77^.

Our results also show that EVs-H1% induced a significant change in the glycolytic capacity of the HUVECs, although it did not enhance cell proliferation in this cell type. These results contrast with other publications where authors demonstrated that EVs promoted vascular endothelial cells angiogenesis via ANXA2^78^, one of the proteins present in EPIC-derived EVs. Considering that glycolysis is the primary energy-producing mechanism in proliferative endothelial cells^79^, these results strongly suggests that epicardial-secreted EVs may induce a glycolytic preconditioning in the targeted endothelial cells, perhaps through the incorporation of glycolytic enzymes enriched in EVs-H1% such as PKM, ENO1 or PGAM1^80–83^. Taken together, these results show that the internalization of EVs cargo triggers an altered metabolism of the receiving cells, indicating the capacity of epicardial-derived cells to affect the cellular environment in both an autocrine and a paracrine manner.

The second part of our work focuses on the extracellular matrix (ECM) derived from the epicardial cells. As previously discussed, EPDCs importantly contribute to the building of the cardiac extracellular component. It is therefore obvious that unveiling the molecular composition of the epicardial-derived ECM is crucial to understanding how cells respond to changes in their microenvironment^62^. So far, most studies into epicardial-secreted ECM proteins are limited to a general expression mapping of a few molecules in the vicinity of the epicardium. Taking advantage of the ECM synthesized by the EPIC line, we have completed the most systematic characterization of epicardial-derived ECM proteins to date.

Using shotgun proteomics, we found representative cardiac ECM proteins in the EPIC secretome. Such proteins were either associated with the basement membrane of the epicardium, such as laminins, FN-1 and several collagens [α1(IV) (COL4A1) and α2(IV) (COL4A2)], or to the interstitial subepicardial matrix, such as collagen α1(I) chain (COL1A1), α2(I) (COL1A2) and α1(III) (COL3A1)^62,84,85^. Remarkably, EPIC ECM contain proteins associated with vascular growth and wound healing phenomena as FN-1, lysil oxidase (LOX), ANXA2, fibulin-1 (FBLN1), matrix Gla protein (MGP), collagen triple helix repeat-containing protein 1 (CTHRC1))^86–92^. This is in accordance with our understanding of the epicardial contribution to embryonic development and adulthood Indeed, 67% of the ECM proteins isolated from mouse embryonic hearts were also found within the EPIC-secreted ECM protein extract, including LAMB1 and 2, FN-1, COL1A1 and 2, FIBLN2, heparan sulfate proteoglycan (HSPG) and EMILIN-1^62,93–96^. Notably, the importance of these components in cardiac morphogenesis has been demonstrated by genetic linkage to congenital heart malformations (106–108), suggesting that EPIC ECM composition is similar to the naïve epicardial-derived ECM.

In this study we also demonstrate that the similarity between Matrigel®’ (the gold standard basement membrane derived ECM gel) and EPIC-ECM is limited to a few protein elements. Prompted by the well-known use of Matrigel for the growth of endothelial cells and vascular structures, we aimed to test EPIC ECM as an in vitro cell growth substrate. Our results confirmed an increase in the proliferation of endothelial cells seeded over EPIC ECM as compared with Matrigel®. Based on these data, we hypothesized that growth effects could result from the effect of both EPIC ECM composition and ECM-bound growth factors and chemokines released in response to the growing cells (e.g. by the partial degradation of the substrate). This interpretation is compatible with the presence of chemokines, such as CXCL12 (a.k.a. stromal cell-derived factor 1, SDF-1), TGFB2, or PDGFB in our EPIC-ECM.

The interactions between EVs and ECM have been studied to decipher their impact in the biodistribution of EVs in various biological contexts^97,98^. EPIC EVs and ECM analytical datasets identify several proteins from both samples that can interact in the extracellular milieu, suggesting a potential interaction between the two components of the EPDCs secretome (EVs and ECM). For example, several studies indicated that heparan sulfate, identified in EPIC ECM, interacts with ANXA2, identified in EPIC EVs, to promote the exosomes uptake in the receptor cells^78^, supporting our results regarding the autocrine effect of EVs on the EPDCs. On the other hand, several studies have also shown that EVs can influence cellular behavior as well as ECM modulation and reorganization^99–101^. In this context, FN, one of the proteins shared by both EPIC EVs and EPIC ECM datasets, is one of the earliest ECM proteins assembled in tissue development^102^, and at the same time, directs EVs to target receptor cells^103^. Importantly, matricellular proteins are thought to be internalized by endocytosis and re-secreted as EVs^101^. This may hint at the existence of an epicardial ECM-EVs interplay in the heart, which may ultimately affect autocrine and paracrine EVs functions.

A critical analysis of our results identifies various limitations in the use of EPIC or similar cells to study the epicardial-derived secretome. The main limitation perhaps is the intrinsic difficulty in validating our findings *in vitro* and *in vivo*. The transient nature of embryonic epicardial and epicardial-derived cells make difficult to obtain enough biological material for -omics approaches. On the other hand, it is also difficult to speculate on the potential effect of EPIC immortalization on the original composition of the EPDCs secretome. We have nevertheless shown that EPIC cells preserve various embryonic EPDCs properties, including their ability to differentiate into cardiovascular cell types, active cell migration, and ECM synthesis and proteolysis^50^. Furthermore, the immortalization of similar types of mesenchymal cells, with high secretory properties, has been demonstrated to be useful in facilitating the production/purification of secreted factors by bypassing limits imposed by the occurrence of senescence (scale-up process) without significantly affecting the composition of their secretome^104,105^.

Altogether, the results of our study provide novel experimental evidence for the essential contribution of epicardium and epicardial-derived cells to the cardiac extracellular environment and open a promising field of study for cardiac repair and regeneration. First, the data presented here has clinical translational potential at different levels. Our data may contribute to using EVs as *bona fide* diagnostic or prognostic biomarkers for cardiac health^39,71,106–108^, considering the promising EVs-based therapies for the repair/regeneration of the diseased heart described during the last decades (reviewed in^35^. On the other hand, the use of biocompatible matrices, alone or in combination with cells and growth factors, remains a plausible strategy for the repair of damaged tissues^109^. Due to its characteristic protein content and its similarities with the embryonic cardiac ECM, the epicardial-secreted ECM described in our study could be of interest for the development of biological scaffolds for reparative therapies. However, all these hypotheses need to be validated both *in vitro* and *in vivo*.

## Supplementary Information

Below is the link to the electronic supplementary material.

Supplementary Material 1

Supplementary Material 2

Supplementary Material 3

Supplementary Material 4

Supplementary Material 5

Supplementary Material 6

Supplementary Material 7

Supplementary Material 8

Supplementary Material 9

Supplementary Material 10

## Data Availability

All TMT data from the experiments performed for EVs characterization available at PRIDE database under accession number PXD059112 (Token: BR6wZn2bzypf). Shotgun data from proteomics experiments performed for ECM characterization are available under request to the corresponding author.
