# Supplementary figures and images for: Revealing the complexity of the epicardial secretome
Source: Sci Rep. 2025 Nov 21;15:41197. doi: 10.1038/s41598-025-24980-y (PMC12639070; doi:10.1038/s41598-025-24980-y)

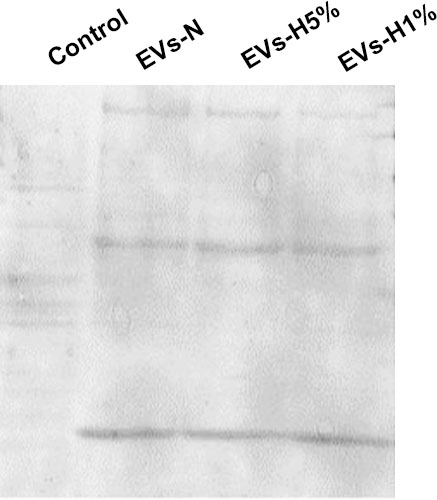

Supplement: Supplementary file 2 — Supplementary Material 2 [file 41598_2025_24980_MOESM2_ESM.tif]

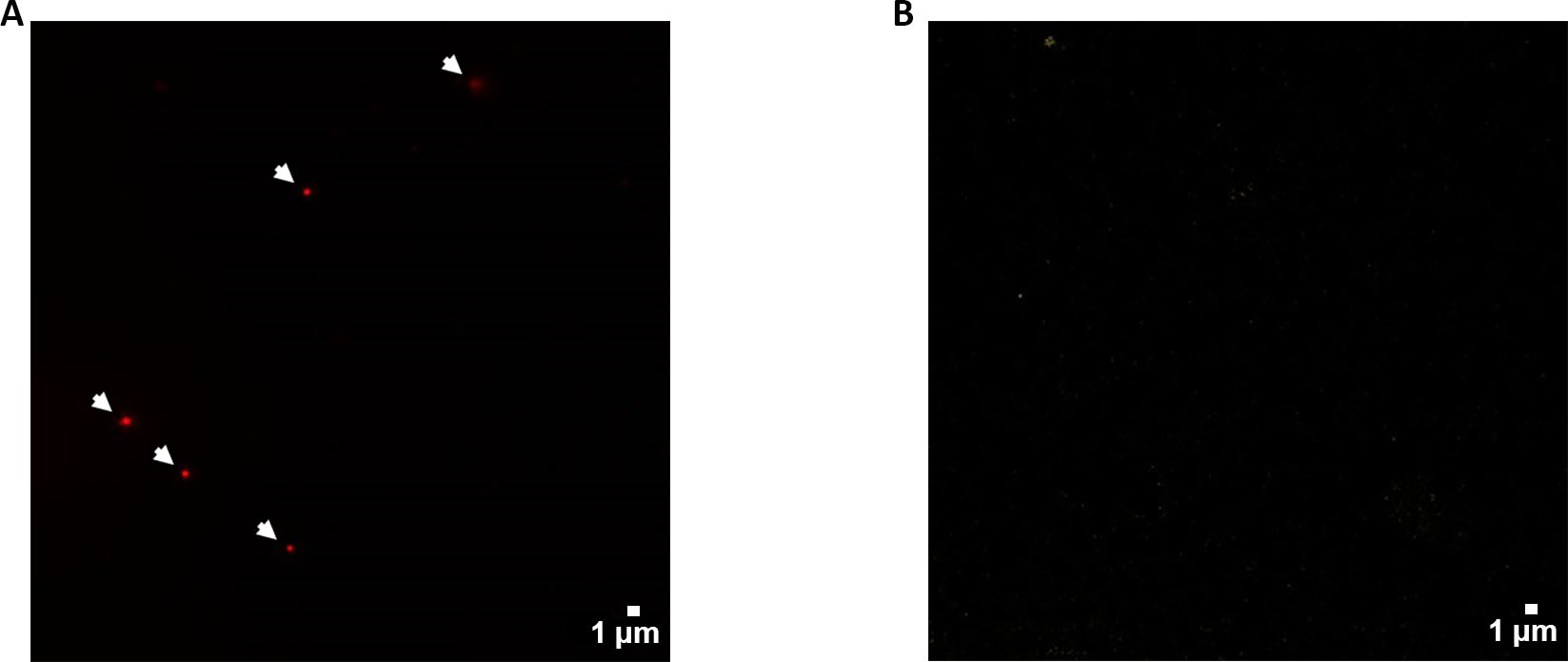

Supplement: Supplementary file 3 — Supplementary Material 3 [file 41598_2025_24980_MOESM3_ESM.tif]

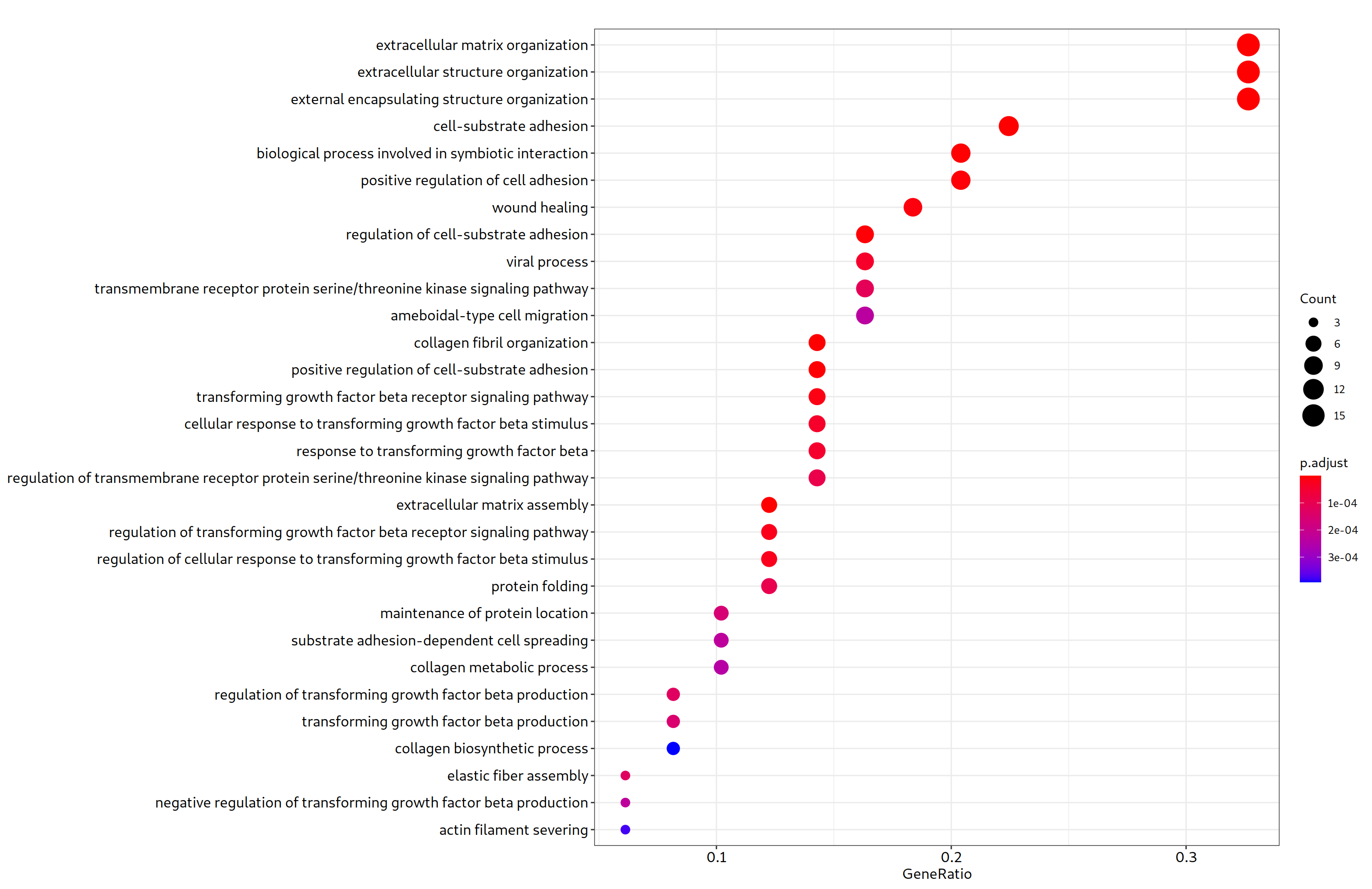

Supplement: Supplementary file 4 — Supplementary Material 4 [file 41598_2025_24980_MOESM4_ESM.tif]

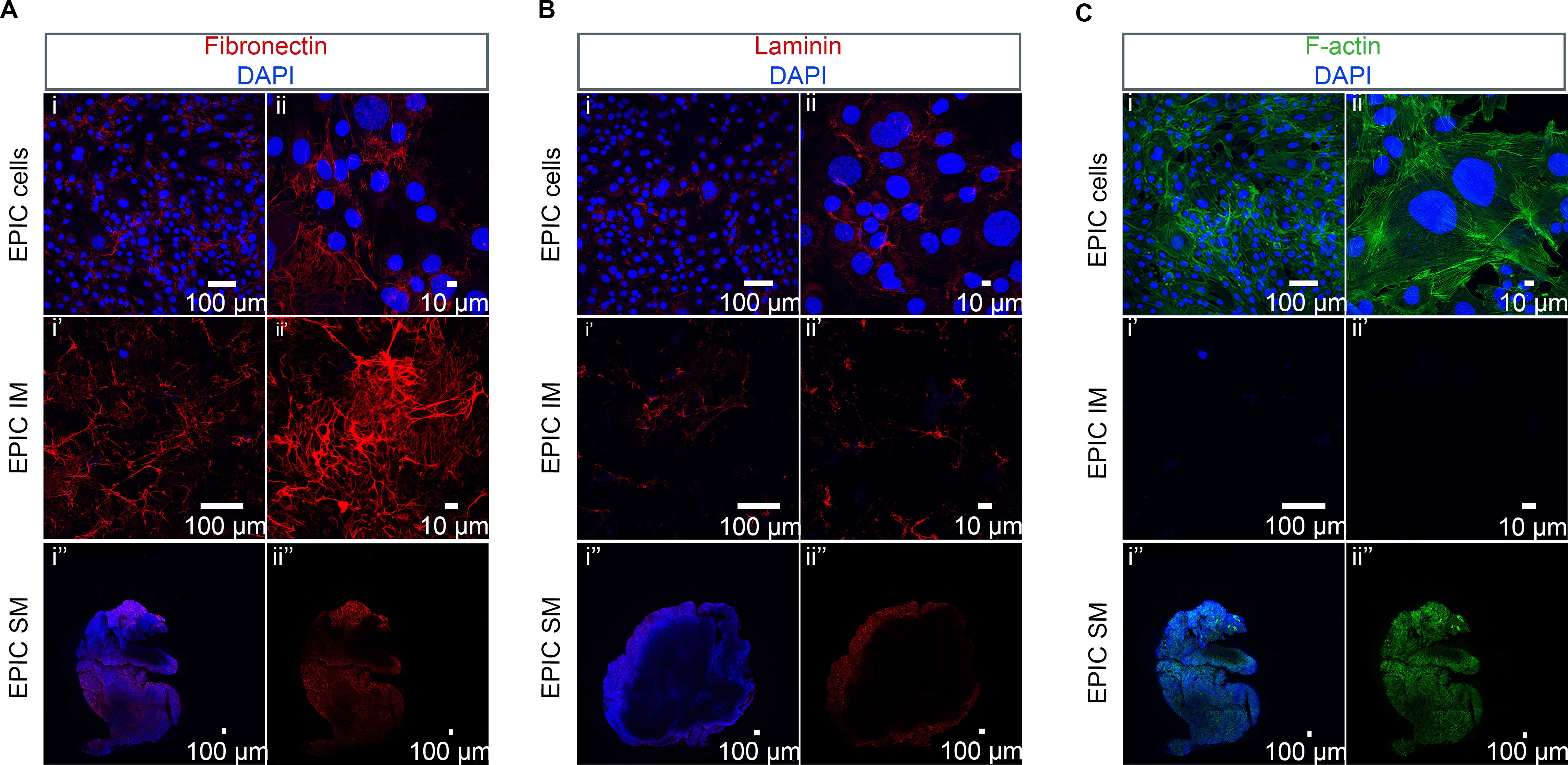

Supplement: Supplementary file 5 — Supplementary Material 5 [file 41598_2025_24980_MOESM5_ESM.tif]

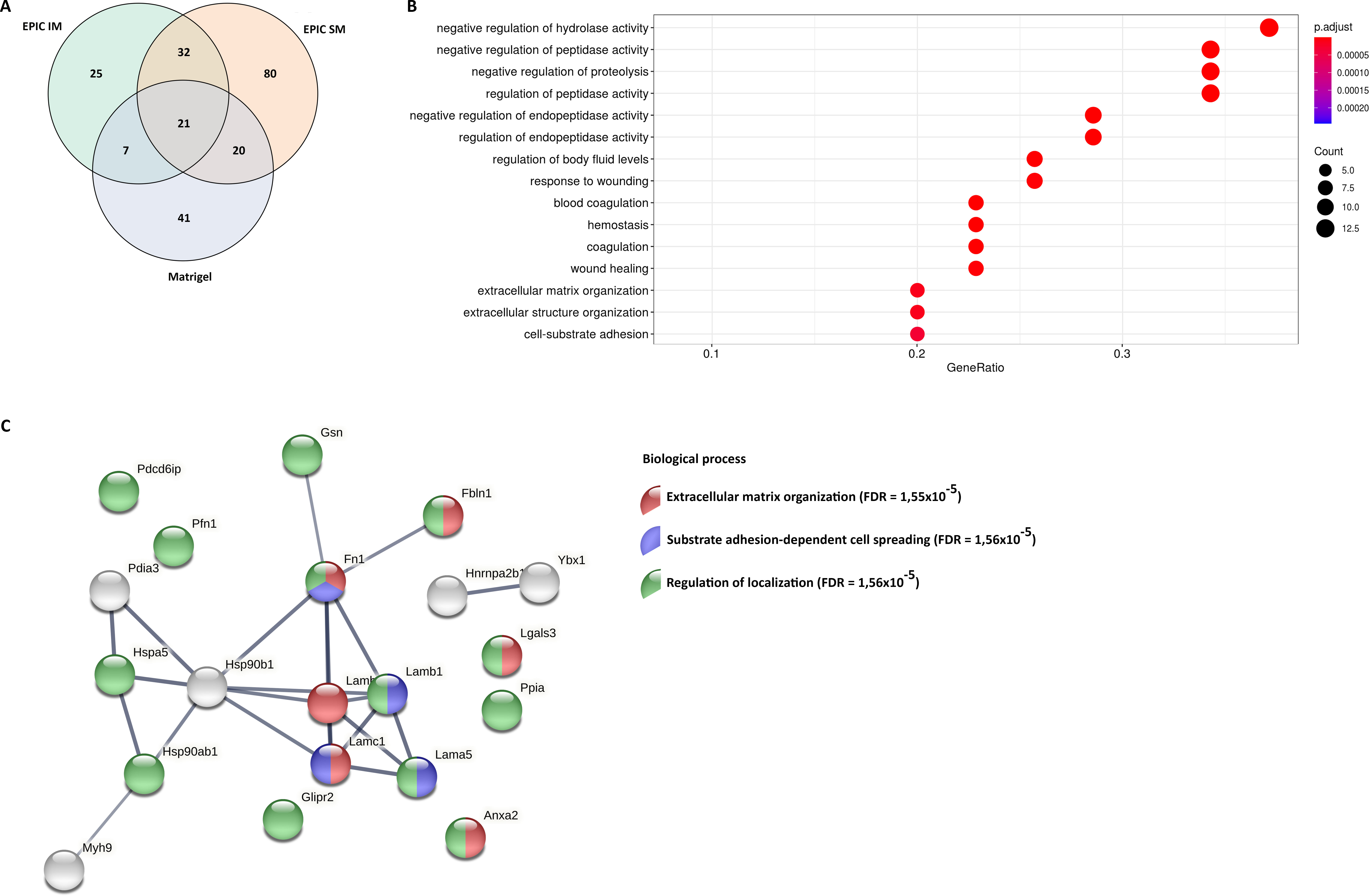

Supplement: Supplementary file 6 — Supplementary Material 6 [file 41598_2025_24980_MOESM6_ESM.tif]

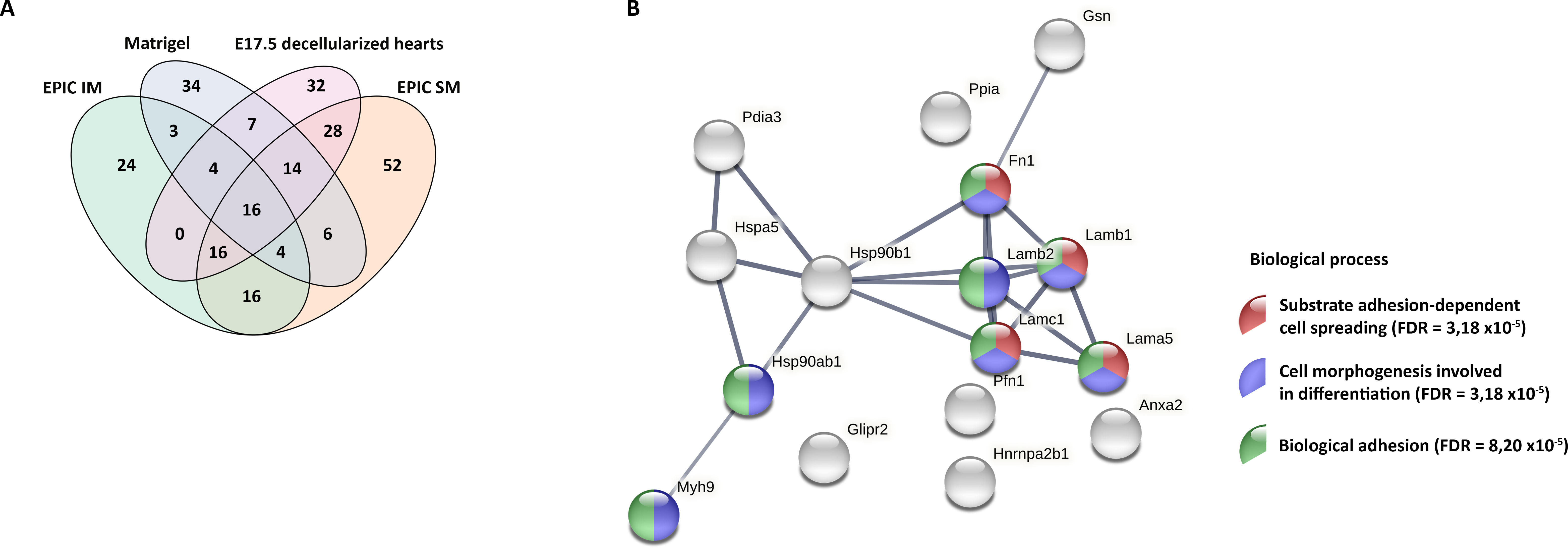

Supplement: Supplementary file 7 — Supplementary Material 7 [file 41598_2025_24980_MOESM7_ESM.tif]

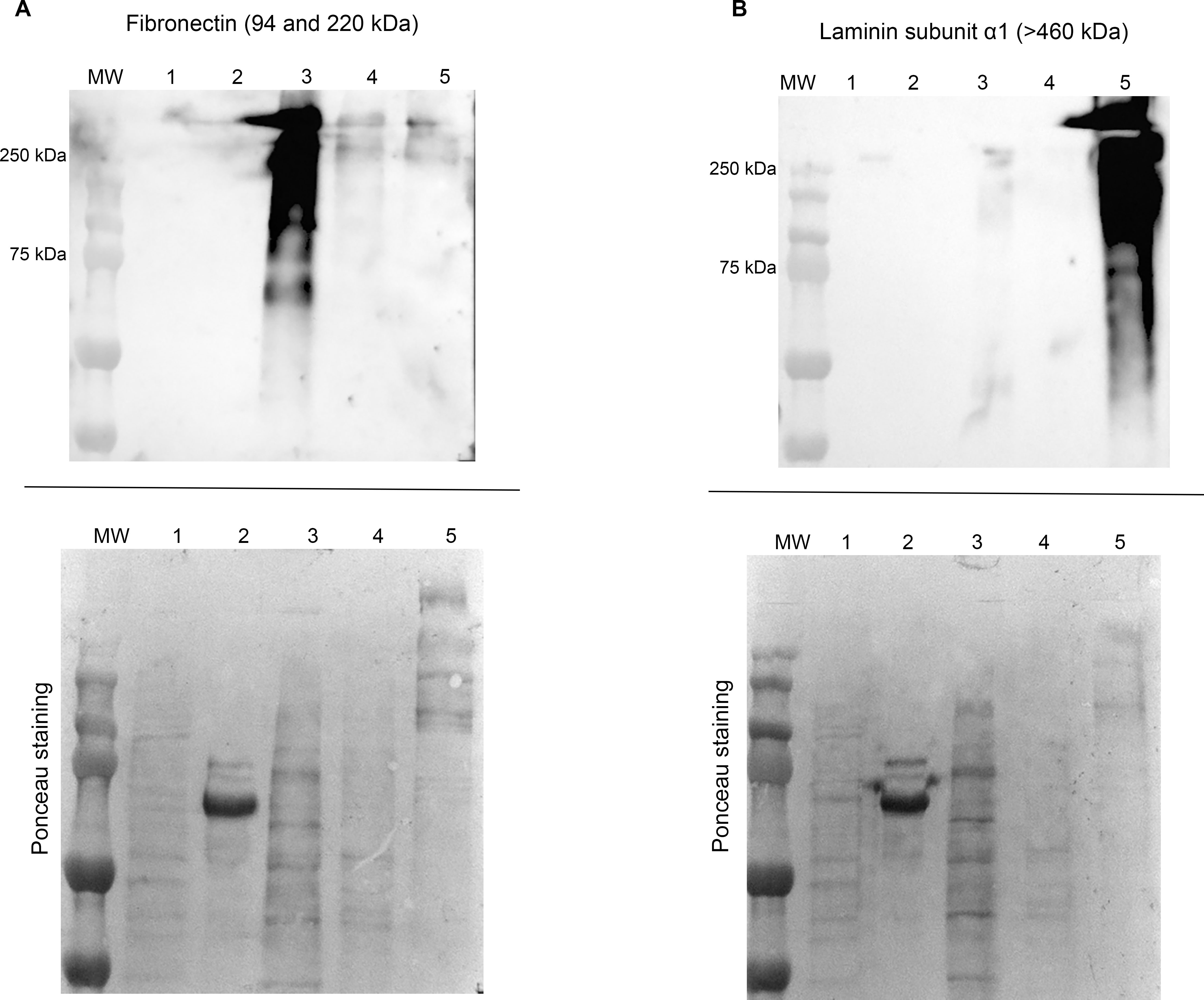

Supplement: Supplementary file 8 — Supplementary Material 8 [file 41598_2025_24980_MOESM8_ESM.tif]
